# Procrastination during the COVID-19 Pandemic: A Scoping Review

**DOI:** 10.3390/bs12020038

**Published:** 2022-02-06

**Authors:** Alejandro Unda-López, Gabriel Osejo-Taco, Andrea Vinueza-Cabezas, Clara Paz, Paula Hidalgo-Andrade

**Affiliations:** Escuela de Psicología y Educación, Universidad de Las Américas, Quito 170124, Ecuador; ruben.unda@udla.edu.ec (A.U.-L.); gabriel.osejo.taco@udla.edu.ec (G.O.-T.); andrea.vinueza@udla.edu.ec (A.V.-C.); clara.paz@udla.edu.ec (C.P.)

**Keywords:** academic procrastination, lockdown, procrastination, COVID, quarantine

## Abstract

Procrastination involves voluntarily or habitually delaying unpleasant tasks for later. It is characterized by short-term benefits and long-term costs. The COVID-19 pandemic set specific circumstances that may have influenced procrastination behavior. This scoping review identified the existing peer-reviewed literature in English or Spanish about procrastination during the COVID-19 pandemic (January 2020 to April 2021) in six electronic databases. To conduct the review, a five-step methodological framework, as well as established PRISMA guidelines, was followed. A total of 101 articles were found. After removing duplicates and reviewing the articles, only 13 were included in the review. Findings indicate that procrastination was studied mostly in academic contexts in various parts of the globe. Procrastination behavior was related to anxiety, distress, time management, self-control, and other variables. There is limited information about interventions to prevent or decrease procrastinating behaviors in the context of confinement or in the living conditions generated by the pandemic. Future research should consider how procrastination evolved during the pandemic using longitudinal methodologies. Individual differences related to procrastination also should be identified, and the evaluation of the efficacy of existing interventions is still needed. This information might help in the creation of appropriate interventions that target detrimental procrastination behaviors.

## 1. Introduction

In December 2019, the presence of a severe respiratory infection was detected in China. The disease, named “coronavirus disease 2019” (COVID-19) [1], spread rapidly outside of China; by 11 March 2020, it was declared as a pandemic by the World Health Organization Director, Dr. Tedros Adhanom [2]. To preserve the health of the population and avoid contagions, several countries adopted restriction measures. Mostly, those measures promoted closing country borders, home confinement, and social distancing [3]. All these restrictions affected the way people developed their daily activities such as working, studying, and leisure. These activities changed abruptly to remote settings, helped by the Internet and communication technologies.

The changes in daily activities provoked the presence of emotional distress [4], amplified also by the uncertainty of living in a pandemic [5]. To deal with these situations, people engaged in activities, mostly in front of screens [6] and, in some cases, procrastinated on some other compulsory tasks.

Procrastination is a psychological term that involves voluntarily or habitually delaying unpleasant tasks for later; it is characterized by short-term benefits and long-term costs [7,8,9]. Procrastination has been studied in different contexts, including academic [9,10,11], work [12], daily life [13], and health [14].

Academic procrastination refers to deliberate and unnecessary delay in completing academic tasks for no reason. It leads to painful feelings and negative learning experiences [9,11]. Several studies suggest that academic procrastination is a consequence of a self-regulation deficit in students [15,16]. Likewise, evidence indicates that personality traits, such as neuroticism and extraversion, are associated with procrastination [17].

Furthermore, procrastination at work is defined as delaying work activities by performing non-work-related actions during working hours, with no intention of harming the employer, employee, workplace, or customer. There are two subtypes of procrastination at work: (1) soldiering, which refers to the avoidance of performing work tasks for more than one hour per day without intent to harm others, and (2) cyberslacking, which is when employees may give the impression that they are working on their computers, but they may be performing other personal activities such as shopping online, using social networks, etc., incurring high costs to companies [12]. In the workplace, organizations have concerns about employees sometimes engaging in non-work-related activities during working hours. In a study conducted by Metin et al. [18] using a sample of 380 white-collar full-time employees in The Netherlands, the authors found a negative correlation between work engagement and procrastination; this means that people with elevated levels of commitment clearly did not spend much time on non-work-related activities during working hours. Hence, procrastination and performance were negatively related.

On the other hand, procrastination of everyday life is the extent to which people perform routine life tasks on time or late, differing according to personal time orientation [13]. This domain can include delaying various kinds of activities, such as filing income tax returns, performing household duties, engaging in hobbies on a regular basis, visiting parents, returning a phone call, writing an e-mail, or meeting friends [19]. In a study in the US, carried out by Ferrari et al. [20] using three different adult samples, findings suggest that procrastination of everyday tasks can lead to problems with clutter in older adults. This is important because these problems related to clutter can reduce a person’s general satisfaction with life.

Meanwhile, procrastination of health behavior is a delay in seeking treatment or postponing treatment [14]; for example, scheduling a doctor’s appointment or implementing wellness behaviors. In a study conducted by Sirois et al. [21] using a sample of 122 university students in Canada, results showed that the relationship between procrastination and health is mediated by health behaviors. In addition, the higher levels of stress experienced due to this behavioral style increase the risk of disease. In fact, procrastinating can further negatively impact health status.

Everyone tends to procrastinate at various times in their lives. Some people tend to procrastinate most of the time, and others tend to procrastinate in specific situations [15]. Several variables related to procrastination have been identified, such as academic anxiety, self-handicapping, and COVID-19 fear in students. An explanation is that students could only study online given the imposed lockdowns, thus providing a more favorable context to procrastinate [8,10,11,22,23,24,25,26,27,28,29].

The COVID-19 pandemic was an unprecedented event that changed the daily activities of most of the world’s population [7]. There is evidence that procrastination during this period may present positive benefits such as an opportunity to “redefine career goals, rethink relationships, relook at personal philosophies, and re-explore passions” [30], whereas other studies have analyzed the negative effects including difficulties in distance learning [29], the perception that time is moving faster [28], an increase in Internet use [10], excessive use of social media [26], general distress [23], higher levels of depression, and decreased academic performance [31].

Currently, people are experiencing circumstances that have not been observed in the past, and research related to the effects of the COVID-19 pandemic on procrastination is still in its initial stages. The existing findings are mixed, which suggests the need for a careful review of the literature to attain a holistic understanding of the results, identification of caveats in knowledge, and recognition of existing interventions. Unlike systematic reviews, scoping reviews allow us to examine emerging evidence and provide an overview of it [32]. Thus, for this article’s objectives, a scoping review was a more valid approach.

In this study, we sought to contribute to the existing literature for the development of future theoretical and empirical research. The aims of the current review were the following aspects:

1. To recognize the psychological variables associated with procrastination during the COVID-19 pandemic;
2. to map the contexts in which procrastination was studied;
3. to identify the sociodemographic characteristics of the involved participants;
4. to analyze the changes in the procrastination levels due to the pandemic and whether any type of intervention has been conducted to address it;
5. to identify the instruments used for the study of procrastination.

The results of this review will provide a reference point that will allow suggestions for future research and interventions.

## 2. Materials and Methods

To carry out this scoping review, we followed a five-step methodological framework [33,34], which includes the following steps:

1. identifying the research question;
2. identifying relevant studies;
3. study selection;
4. charting the data;
5. collating, summarizing, and reporting the results.

The research question, objectives, and methods of the scoping were defined before starting the process, and the project is publicly available in ResearchGate. Our purpose was to find out what has been studied regarding procrastination during the COVID-19 pandemic. Indeed, this study aimed to identify the topics studied around procrastination during this unprecedented time. Secondarily, we wanted to explore related mental health variables, contexts (e.g., academic, work, etc.) in which it has been studied, types of studies, instruments for its measurement, levels of procrastination, and interventions to prevent or decrease procrastination.

After defining the scoping review, we specified data sources and search strategies. This research was restricted to peer-reviewed literature from January 2020 to April 2021. We use established search terms and searched each of the following electronic databases: Scopus, Web of Science, PubMed, PsycInfo, Ebsco, and Google Scholar. The search was conducted for the title, abstract, and keywords and included the following terms: procrastinate OR procrastination AND COVID-19 OR COVID OR coronavirus OR SARS-CoV OR pandemic OR lockdown OR quarantine.

Before the initial screening, duplicates were removed. The abstracts of the studies were examined by three members of the research team for eligibility. The inclusion criteria included (1) articles written in Spanish or English, (2) papers published in peer-reviewed academic journals between 30 January 2020 and 20 April 2021, (3) those that described procrastination during the COVID-19 pandemic, and (4) they should be original studies (quantitative, qualitative, or mixed methods studies), letters to the editor, conference abstracts, reviews, and pre-prints. Other grey literature was not included because our aim was to examine peer-reviewed papers to assure the quality of the studies to some extent and make the search as systematic as possible. After this initial screening, discrepancies were discussed with the whole research team. The full texts of the included articles in the first screening were retrieved and reviewed in a second screening, using the inclusion criteria of the first screening.

To chart the data, three members of the research team independently retrieved basic information about the article, as well as more specific aspects of the content of the article such as sociodemographic characteristics of the sample, instruments used to measure procrastination, other variables studied, details about the interventions, type of publication/research, design and methodology used, participants, conclusions, recommendations, and limitations. Then, the results were discussed as a group to identify and overcome any inconsistencies. Finally, to summarize and report the process and the results, we used the checklist of PRISMA extension for scoping reviews (PRISMA-ScR), a 22-item list intended to provide guidance on the reporting of this type of review [35]. The complete checklist is included as a Supplementary File.

## 3. Results

**Figure 1:**
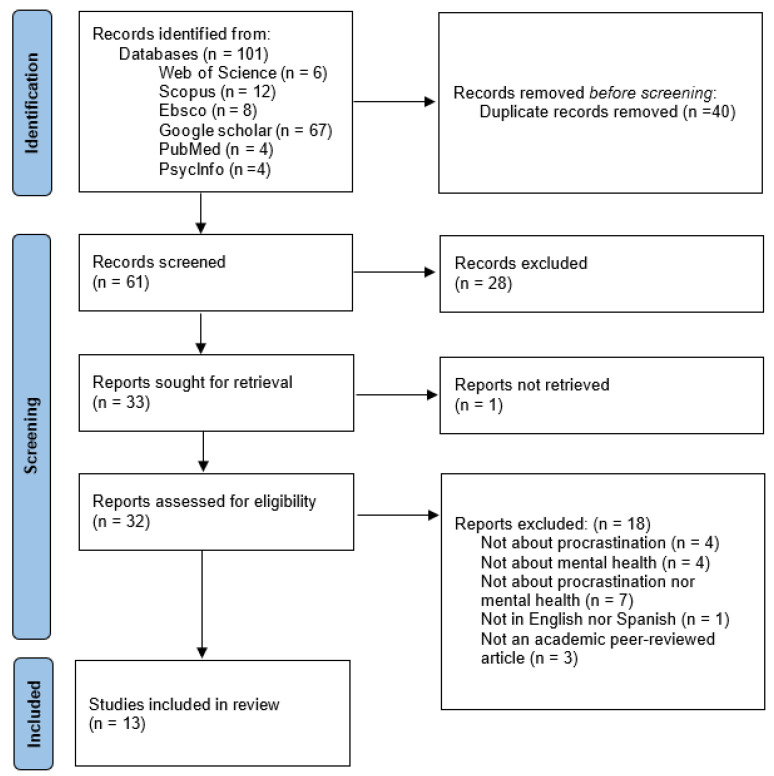
PRISMA flow diagram for source of evidence selection [36].

As seen in Figure 1, the initial search yielded 101 documents, from which 40 duplicates were removed, and 61 articles were assessed. An initial screening was conducted based on inclusion criteria, which resulted in the selection of 33 articles; one study could not be retrieved. Three members of the research team conducted a full-text screening for the remaining 32 articles. A total of 13 articles fully met the inclusion criteria, 2 of which were pre-prints [23,25].

The information provided by these 13 articles is described by each variable of interest as follows. Most of the reviewed studies used a quantitative methodology [8,10,11,22,23,24,25,26,27,28,29], one used a mixed methodology [37], and one used a qualitative methodology [38]. Of the 13 articles included, 12 studies used a cross-sectional methodological design. Only one article presented a longitudinal study, which had a follow-up two months after the first data collection to investigate how resilience, distress, and fear toward COVID-19, among other factors, changed during the pandemic [23]. Table 1 describes the main information of the articles included. The same table also includes an assessment of the articles’ quality. This assessment was developed under the scoring system of systematic review tool “QualSyst”, a 14-item checklist for quantitative studies, and another 10-item checklist for qualitative studies. The appraisal score depends on the degree to which specific criteria are met, and the total score is divided by the number of applicable items, where “yes” equals a score of 2, “partial” equals to a score of 1, and “no” equals to a score of 0 [39]. In the 13 included studies, scores between 1.18 and 1.81 were determined out of a maximum score of 2. These results support the inclusion of the selected material, as it is suggested that studies appraised below a score of 0.75 should be excluded [39].

### 3.1. Variables Related to Procrastination

Two studies found that anxiety was positively correlated with procrastination and presented procrastination behavior as a cause of general distress due to the pandemic [11,25]. Additionally, academic anxiety and self-handicapping [25] were found to be positively correlated to procrastination. Other related variables were birth order and permissive parenting style [27], the ability to adapt to virtual methodology [29], academic performance [10], mental well-being [23,38], the psychoemotional state generated due to forced social distancing measures [8], a person’s neuroticism [24], lack of motivation, and stress [37].

Other variables may predict procrastination such as hedonic use of social network sites, job escapism, socializing [26], or because of lockdown adaptations [28]. On the other hand, it was found that procrastination negatively correlated with several variables such as digital competence and digital literacy [22], high perceived competence, time management skills, meta-cognition [37], hardiness [25], parents’ education, high self-control [27], and a person’s openness, conscientiousness, and extraversion [24].

### 3.2. Sociodemographic Characteristics of the Samples

Regarding the sociodemographic characteristics of the samples in the 13 included empirical studies, participants’ characteristics varied, although most of them were students and workers aged between 14 and 60 years old. Age was found in one study to be positively related to procrastination. Specifically, a study showed that students aged 23 and older have higher levels of academic procrastination than students between the ages of 17 and 19 [11]. Additionally, gender was a significant variable in one study where male students were found to exhibit a higher presence of procrastination behaviors than female students [10].

The geographical regions of the empirical studies varied: two studies were from China [24,25], two were conducted in Russia [22,29], and two in Turkey [10,11]. Other studies included in this scoping review were conducted on Algeria [38], Austria [37], Greece [26], Indonesia [27], Pakistan [28], and Ukraine [8]. One study presented results from several countries, including Brazil, Colombia, Germany, Israel, and Norway [23].

### 3.3. Context and Environment

Procrastination was mostly studied in academic contexts. In total, 10 studies included a sample of university and high school students, with variables related to the academic environment having an impact on procrastination, such as internet consumption [10,26], task aversiveness and fear of failure [37], sleep patterns [28], parenting styles [27], and anxiety [11,25]. On the other hand, four of the articles studied procrastination in the same academic context but focused on how procrastinating behavior affected the learning process in distance education amongst students and schoolteachers [8,23,30,38].

### 3.4. Instruments Used to Measure Procrastination

All the reviewed articles measured the levels of procrastination during the pandemic. Table 1 indicates the instruments used by each study and shows that three instruments were commonly used—namely, the Academic Procrastination Scale [40], the Tuckman’s Procrastination Scale [41], and the General Procrastination Scale [42]. Other instruments employed were the Procrastination Questionnaire for Students [43] and the Procrastination Academic Scale for Students [44]. Five studies also implemented questionnaires designed specifically for their studies [23,26,28,29,38].

### 3.5. Intervention Strategies

The reviewed literature did not present strategies focused on lowering or changing the levels of procrastination; two articles, however, made suggestions on how to reduce it but neither implemented nor measured the suggested strategies. The first study observed the effect of physical activities during the lockdown and concluded it could decrease negative feelings associated with anxiety and depression; it presented a weak correlation with lower levels of procrastination [11]. The second strategy aimed to lower procrastination habits and to increase levels of self-control by creating organizational skills, setting goals, and promoting healthy habits [27].

**Table 1:** General information of selected articles.

Article Title	Author (year)	Type	Country	Objectives	Instruments for Measuring Procrastination	Conclusion and Recommendations	Quality Assessment Based on “QualSyst” [39]
COVID-19 fear in sports sciences students and its effect on academic procrastination behavior	Biricik and Sivrikaya [11]	Scientific article	Turkey	To examine the COVID-19 fear levels of the students of the faculty of sports sciences and their academic procrastination behavior in terms of various variables and to determine the correlation between them.	Academic Procrastination Scale [40]	There was a weak positive correlation between COVID-19 fear and academic procrastination behavior. Students who worked out during the pandemic had less fear of COVID-19; however, this had a weak effect on reducing academic procrastination behavior. Higher education institutions should provide aid for students to overcome negative emotions and improve academic performance.	1.55
Distance learning during the corona-lockdown: some psychological and pedagogical aspects.	Valieva [29]	Conference Paper	Russia	To summarize and analyze factors that, to a certain extent, influenced the effectiveness of distance learning for educators.	Author’s questionnaire of irrational procrastination	The teacher’s ability to procrastinate can play a certain role in the issue of adjustment to rapidly changing conditions in the distance mode; other times, quick decision-making may be crucial.	1.22
Effects of COVID-19 pandemic and lockdown on lifestyle and mental health of students: A retrospective study from Karachi, Pakistan	Ali et al. [28]	Scientific article	Pakistan	To investigate the correlations between changes in sleep pattern, perception of time, and digital media usage. To explore the impact of these changes on the mental health of students of different educational levels.	Author’s questionnaire	Findings indicate the decline in the mental health of students due to the lockdown. Longitudinal studies on the subject are needed.	1.73
Examining the relationship between academic procrastination behaviors and problematic Internet usage of high school students during the COVID-19 pandemic period	Tezer et al. [10]	Scientific article	Turkey	To examine the relationship between academic procrastination behaviors and problematic Internet usage of high school students during the COVID-19 Pandemic.	Academic Procrastination Scale [40]	Results show that academic procrastination behavior and problematic internet usage have a positive correlation.	1.64
A preliminary study of online learning: the influence of the class approaches and the personality of students	Sun et al. [24]	Scientific article	China.	To answer these questions (1) Will different formats of online courses generate different learning effects? and (2) What kinds of students are best fitted in each of these approaches?	Tuckman’s Procrastination Scale [41]	Procrastination is negatively correlated with a person’s openness, conscientiousness, and extraversion; however, it is positively correlated with a person’s neuroticism. More studies are needed to design a self-reflection scale that could be used for online learning to improve the reliability and validity of the data.	1.73
Identifying resilience factors of distress and paranoia during the COVID-19 pandemic	Mækelæ et al. [23]	Pre-print.	Brazil, Colombia, Germany, Israel, and Norway	To study resilience, a successful adaptation despite risk and adversity, in five countries: Brazil, Colombia, Germany, Israel, and Norway.	Authors questionnaire	Thriving, keeping a regular schedule, engaging in physical exercise and less procrastination served as factors protecting mental well-being. Procrastinators had a higher score on general distress than participants who spent no or little time procrastinating.	1.36
Influence of poor digital competence on procrastination of university teachers	Kosycheva et al. [22]	Scientific article	Russia	To describe the possible ways of evaluating digital competence and digital literacy of university teaching staff and underline the importance of university teachers’ ICT skills development.	Tuckman’s Procrastination Scale [41]	Procrastination in teachers is correlated to working conditions; digital competence and digital competence beliefs; fear of failure and tasks aversiveness.	1.18
Learning during COVID-19: the role of self-regulated learning, motivation, and procrastination for perceived competence	Pelikan et al. [37]	Scientific article	Austria	To investigate differences in students who perceived themselves as high vs. low in competence with respect to these constructs.	Passive procrastination adapted from Procrastination Questionnaire for Students [43]	Students who experienced themselves as highly competent use SRL strategies procrastinated less.	1.60
Academic anxiety and self-handicapping among medical students during the COVID-19 pandemic: a moderated mediation model	Jia et al. [25]	Pre-print	China	To examine the mediating role of procrastination and moderating role of hardiness in the association between academic anxiety and self-handicapping during the COVID-19 pandemic.	The General Procrastination Scale [42]	Academic anxiety was positively correlated with procrastination and self-handicapping, and negatively correlated with hardiness. Procrastination was positively correlated with self-handicapping and negatively correlated with hardiness; hardiness was negatively correlated with self-handicapping. Furthermore, procrastination partially mediated the relationship between academic anxiety and self-handicapping, and both the effects of academic anxiety on self-handicapping and the mediating effect of procrastination were moderated by hardiness.	1.45
Motives and consequences of social network sites: teachers in Greece a case study	Gougas and Malinova [26]	Scientific article	Greece.	To evaluate the hedonic use, utilitarian use, socializing, procrastination, job escapism, and work productivity of the specific professional team from the use of social network sites (SNSs).	Authors’ questionnaire	Hedonic use of SNS is correlated with procrastination to analyze the relationship between the motives for use of SNSs.	1.18
Academic procrastination during the COVID-19 pandemic: the control, parenting style, and family factors	Rahdadella and Latifah [27]	Conference Paper	Indonesia	To identify individual characteristics, family characteristics, parenting styles, self-control, and academic procrastination and analyze the influence of individual characteristics, family characteristics, parenting styles, and self-control towards academic procrastination in undergraduate students.	Procrastination Academic Scale for Students [44]	The birth order and mother’s permissive parenting style positively affected academic procrastination. Additionally, the father’s education and the student’s self-control had a significantly negative effect on academic procrastination. It is important to improve self-control and not apply a dominant permissive parenting style, to prevent procrastination.	1.81
The nature of the manifestation of procrastination, level of anxiety and depression in medical students in a period of altered psycho-emotional state during forced social distancing because of pandemic COVID-19 and its impact on academic performance	Romash [8]	Scientific article	Ukraine	To investigate and evaluate the level of anxiety, depression, and the nature of the manifestation of procrastination in medical students Faculty of Medicine and the Faculty of Training Foreign Citizens of Ivano-Frankivsk National Medical University in a period of altered psychoemotional state during the period of forced social distancing and its impact on academic performance.	The General Procrastination Scale [42]	Procrastination occurs in the surveyed medical students in the period of altered psychoemotional state during forced social distancing. Results show that in the group with low procrastination, the success rate is higher than in groups with medium and high procrastination.	1.45
The psychological and behavioral side-effects of coronavirus outbreak (COVID-19) on the Algerian researchers’ scientific work and academic plans: The case of master two and PhD students	Khiari and Khiari [38]	Scientific article	Algeria	To investigate thoughts, feelings, and perspectives of 202 male and female Master Two and PhD students about how the lockdown impacted their research and on what levels.	Author’s questionnaire	Coronavirus crisis and the quarantine have negative effects on their mood, spirits, and psychological well-being, which leads to procrastination and loss of motivation.	1.60

## 4. Discussion

The general distress generated by confinement measures and social isolation may influence procrastination behaviors. The present scoping review aimed to identify articles that studied procrastination between January 2020 and April 2021, the initial stages of the COVID-19 pandemic. Specifically, we looked for changes in procrastination levels, the psychological variables associated with procrastination, the contexts, the sociodemographic characteristics of the participants involved in the studies, and the interventions used or proposed to reduce procrastination.

During the pandemic, there were several negative effects on mental health. Confinement, a protection measure against COVID-19, forced people to adapt to novel lifestyles that may have contributed to procrastination behavior. Findings suggested that the recorded presence of procrastinating behavior may come because of the confinement and lockdown adaptation, as indicated in previous studies [45].

In academic contexts, students experienced greater anxiety, fear, stress, and health concerns due to the pandemic and confinement [11,28]. A study revealed that the problematic use of the Internet, on top of a lack of time management in students during this period, contributed to increased procrastination behaviors on their mandatory activities [10]. Consequently, this significantly affected the students’ performance and caused more stress. Regarding the teachers’ experiences, it was found that the change to virtual modality caused some problems related to digital competencies [22]. The groups that had more difficulties were the older teachers, with issues such as technical problems with communication, more time dedicated to the preparation for classes, and a lack of in-person communication and exchange of emotions with the students, highlighting that the capacity for procrastination could be due to a matter of adjustment to the new circumstances that were being experienced [29].

Another finding of this review was the association of parenting styles with procrastination. Parenting style refers to how parents treat, communicate, discipline, monitor, and support their children [27]. Results indicate that permissive parenting styles correlate positively with procrastination. Specifically, academic procrastination could increase when parents are less demanding and have no hope for achievement with their children. This relationship is more evident in late adolescence since they need a supportive environment in which they can establish priorities and goals [27].

Regarding the instruments used to measure procrastination, the Academic Procrastination Scale [40], the Tuckman’s Procrastination Scale [41], and the General Procrastination Scale [42] were used in two studies each. The use of self-made questionnaires to measure the incidence of procrastinating behaviors was identified in 5 of the 13 studies [23,26,28,29,38]. The use of several tools in the measurement of procrastination does not allow for a comparative analysis of the studies.

Information on intervention strategies was analyzed. Only two of the included articles suggested interventions intended to reduce procrastinating behavior, although outcomes of these strategies were not analyzed. The first study found that physical exercise can reduce the levels of anxiety and depression, having a weak mediating effect on procrastination [11]. Another study conducted by Rahdadella et al. [27] suggested generating organizational skills, setting goals, and promoting healthy habits, to increase levels of self-control, a psychological factor found to have a moderating effect on procrastinating behaviors. Intervention strategies aimed to modify behaviors related to higher levels of procrastination rather than directly attempting to modify procrastinating behaviors. As in our results, a previous literature review [15] showed limited information regarding intervention strategies for procrastination behavior. Therefore, this area still needs to be further studied. The interventions in the mentioned review highlighted strategies that focus on a therapeutic treatment to intervene in procrastinating behavior, therapeutic prevention focusing on the negative effects of procrastination, and other non-therapeutic methods to decrease procrastinating tendencies in students [15].

### Limitations

The reviewed literature presented several limitations that restricted the depth analysis of the evidence. The included studies were conducted in several countries, not only English-speaking countries, which gave us the possibility of exploring different realities. However, only a multicentric study included a Spanish-speaking country [23]. Most of these studies used a cross-sectional methodology, which did not allow the identification of change in procrastination, one of our initial objectives. In addition, most articles did not include intervention strategies, and the ones mentioned were not proven in the studied population. It was also observed that most of the reviewed articles studied procrastination in academic and work-related contexts, so it was not possible to study subjects related to procrastination in other contexts. These issues presented a limitation on the depth of our analysis and desired results. Future studies could focus on intervention strategies for procrastination in the academic and occupational context may target mental health issues and community support, as these factors contribute to the presence of procrastination [15,27], as well as the consequences brought by the pandemic such as isolation, health concerns, and need of adaptation [11,27].

Several limitations emerged regarding the review process. First, grey literature, such as government documents, thesis, and other non-academic documents, was not considered, and only studies written in English or Spanish were analyzed. Finally, the sources of evidence were limited to those published between 30 January 2020 and 20 April 2021. Studies in the literature published after that date may present new findings and useful information that was not included in the present review. Given the dates of our search, published articles might have been initial approximations to the study of procrastination during the pandemic and might be basic in their methodology to provide information on the matter. Additionally, the studies did not consider variations in the development of the pandemic in different countries.

## 5. Conclusions

The present scoping review aimed at identifying the existing evidence regarding procrastination during the initial stages of the pandemic. The literature showed that procrastination and procrastinating behavior were influenced by several aspects caused by the COVID-19 pandemic. Most research was conducted in academic and work-related contexts, where variables related to procrastination were stress, anxiety, general distress, time management skills, self-control, technology use, etc. Interventions presented in the reviewed material did not focus on modifying procrastination or procrastinating behavior but to lower procrastination through exercise and creating healthy habits. No further analysis was conducted regarding data obtained during the studies given the variability in tools implemented in the reviewed material.

Furthermore, several needs regarding future studies of procrastination were identified. There is limited information about interventions that can be carried out to prevent or decrease procrastinating behaviors in the context of confinement and living the consequences generated by the pandemic. These intervention strategies could target mental health issues, health concerns, and social support. The use of a common scale to measure procrastination behavior and levels is needed to compare results. Other researchers should also consider longitudinal methodologies to capture the changes in levels and procrastination behaviors. Future studies need to address individual differences that may influence the levels of procrastination such as personality, culture, resources, and opportunities, as well as how the pandemic may affect a population in different contexts.

## Data Availability

The study protocol is also accessible through ResearchGate (https://www.researchgate.net/project/Procrastinacion-durante-la-pandemia-por-COVID-19-un-scoping-review) (accessed on 20 January 2022). All other information is included in the article.

## Supplementary Materials

The following supporting information can be downloaded at: https://www.mdpi.com/article/10.3390/bs12020038/s1, Figure 1: PRISMA flow diagram for source of evidence selection; Table 1: General information of selected articles; Preferred Reporting Items for Systematic reviews and Meta-Analyses extension for Scoping Reviews (PRISMA-ScR) Checklist.
